# Crowd oil not crude oil

**DOI:** 10.1038/s41467-019-09685-x

**Published:** 2019-04-30

**Authors:** Roland Dittmeyer, Michael Klumpp, Paul Kant, Geoffrey Ozin

**Affiliations:** 1 Institute for Micro Process Engineering (IMVT), Karlsruhe Institute of Technology (KIT), Hermann-von-Helmholtz-Platz 1, Eggenstein-Leopoldshafen, 76344 Germany; 2 Department of Chemistry, University of Toronto, 80 St. George Street, Toronto, M5S 3H6 Canada

**Keywords:** Heterogeneous catalysis, Carbon capture and storage, Energy and behaviour

## Abstract

Climate change represents an existential, global threat to humanity, yet its delocalized nature complicates climate action. Here, the authors propose retrofitting air conditioning units as integrated, scalable, and renewable-powered devices capable of decentralized CO_2_ conversion and energy democratization.

## Introduction

**Fig. 1:**
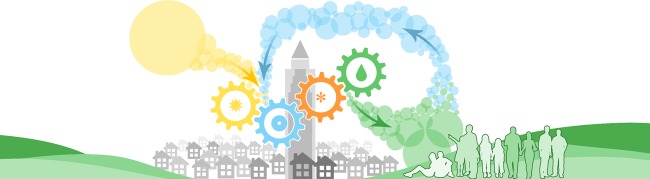
The vision of crowd oil. Renewable oil wells, a distributed social technology, whereby people in homes, offices and commercial buildings all around the world, collectively harvest renewable electricity and heat and use air conditioning and ventilation systems to capture CO_2_ and H_2_O from ambient air and convert it, by chemical processes, into renewable synthetic oil—crowd oil—substituting for non-renewable fossil-based oil—a step towards a circular CO_2_ economy for the benefit of all

Imagine the renewable-electricity-powered air conditioning system in your house, apartment or office at work, besides functioning for cooling and heating, being adapted to capture carbon dioxide and water from the air. Imagine the water and carbon dioxide thus collected converted into renewable hydrocarbon fuels using existing technology and thereby creating personalized, localized and distributed, synthetic oil wells (see Fig. 1). As an alternative to legacy fossil fuel, these oil wells can be tapped, shared and stored, with the option for the property owner to receive payment for any excess fed into a renewable oil grid. Envision this model adopted globally and collectively. This could have a significant impact on the carbon dioxide load emitted into the atmosphere while safely storing available renewable electrical energy and heat in the form of high-energy-density chemical fuel, rather than in pressurized underground carbon dioxide reservoirs with a chance of leakage. As an illustration of the greenhouse gas reduction potential and renewable synthetic oil production capacity of the envisioned approach, a preliminary technical analysis for three practical cases, the Frankfurt Fair Tower office building, a typical grocery store and low-energy houses, is reported. This analysis impressively demonstrates that air conditioning systems in place if equipped with the appropriate technology could indeed capture a very significant amount of carbon dioxide. The envisioned model of “crowd oil” from solar refineries, akin to “crowd electricity” from solar panels, enables people to take control and collectively manage global warming and climate change, rather than depending on the fossil power industrial behemoths. The environmental, economic and social consequences of such a distributed renewable oil well technology should contribute to the practical realization of chemical fuels from carbon dioxide as feedstock in a circular sustainable economy of the future.

## Need for change

Georgius Agricola (1494–1555), German mineralogist and metallurgist, was the first to propose that fossil fuels originate from the remains of plants and animals subjected to heat and pressure in the earth’s crust over millions of years.

Fossil fuels, our natural energy legacy, that provide around 85%^1,2^ of the current global energy supply, are comprised of coal, oil and natural gas. These fuels first powered the Industrial Revolution of 1760–1840 and continue today unabated, despite the transition to renewable forms of energy. Today, approximately half of the earth’s crude oil reserves, two thirds of the earth’s gas reserves and three quarters of the earth’s coal reserves remain underground^3^. At the current rates of consumption, it is projected that the depletion of these reserves will occur within the next decades to centuries^1–4^. However, it may not even take this long for the unabated use of our fossil fuel inheritance to cause severe health problems of millions of people due to suffocating heat, major damage of the natural and built environment by more frequent flooding, thunderstorms, drought, and other catastrophes, and escape or forced resettlement of the population of whole regions or even countries, all caused by the greenhouse gas effect^5–8^. As recently outlined by the Intergovernmental Panel on Climate Change (IPCC)^6^, only a fast transition of world’s economy to an economy with net zero and later in the twenty-first century even net-negative carbon dioxide (CO_2_) emissions will allow humanity to achieve the goals of The Paris Agreement^9^ of 2015 and thus to maintain earth as a planet with limited negative consequences of man-made climate change.

While implementation of renewable solar and wind energy is on the rise and will gradually help ameliorate greenhouse gas emissions, the rate of the transition to a secure global sustainable energy system may not be rapid enough. This is due largely to financial restraints worldwide and lack of consensus by politicians and may not get off the ground because it requires the cooperation and will of so many people in so many countries.

Notwithstanding the challenges of establishing a unified national or international renewable energy policy, myriad science and engineering projects are under development around the world to accelerate and enable the transition from a non-renewable energy supply to a renewable one. These research projects and industry initiatives focus on many aspects of this endeavour such as new materials and technologies for renewable energy harvesting^10–15^, new topologies and methods for stabilizing power grids^12,16–18^, energy storage^16,19,20^, efficient usage of energy^12,21,22^ and renewable power-based transportation fuels and industrial chemicals mostly from captured carbon dioxide or industrial waste gas streams^14,15,23–27^. The latter involves hydrogen as a potential future fuel but also as an important intermediate for the production of synthetic hydrocarbons.

In practice, these projects can only be realized at a scale capable of significantly influencing the trend of climate change if the largest greenhouse gas-emitting industries agree to adapt to the required changes. However, due to industry’s fixation on short-term financial benefits as main decision criteria, this will likely only happen with government incentives such as cap-and-trade or fee-and-dividend, or if business economics permit and decision makers give approval. In this context, note that fee-and-dividend places a price on all products based on their carbon emissions in production/use, collected and distributed to all citizens equally. The rich will pay more, but the poor will reap benefits, causing people to think twice how to spend their windfall carbon money. It is a contemporary idea where people will decide how to spend their carbon money and decide which companies’ products survive, unless they change, as opposed to the cap-and-trade that lets companies decide how to improve.

## The vision of “crowd oil”

Today, there seems to be little public appetite for large-scale CO_2_ capture and underground storage (CCS) combined with a continued use of fossil energy carriers, mainly due to safety concerns^28^. Even though the public awareness of the need to become a CO_2_-negative society is on the rise, mainly due to the IPCC reports and its news coverage, the option of CCS using air-captured CO_2_ still faces the challenge of not generating a valuable product. To make this happen requires a different approach to provide an economic incentive such as compensation payment financed by taxes or voluntary donations.

Perhaps more likely to gain public support is a project wherein people use renewable electricity and solar thermal or waste heat to collect CO_2_ in order to recycle it into hydrocarbon fuels in their homes, apartments and offices. The products generated in these decentralized synthetic oil wells could be used to replace conventional fossil fuels or stored for later use. This could be, for example, for power generation in fuel cells or micro gas turbines at times when there is a deficit of renewable power supply, but also as a transportation fuel in the future. People could either act as individuals owning themselves the infrastructure needed for achieving the conversion and use the fuels for their own purposes or sell it to the market. However, they could also align with others to share the infrastructure or parts of it and this way make more efficient use of it. They could also join forces regarding the marketing of the fuels or the financing of investments into new infrastructures to produce such fuels. Existing or new companies could be involved as well, for example, operating processes emitting CO_2_ (desirably of non-fossil origin or unavoidable) or larger office buildings where CO_2_ capture could be implemented. These could be connected, for example, to renewable power generation by photovoltaics on the roofs of many single-family homes around as well as to conversion facilities at different sites. Furthermore, collectively or privately owned and operated local power grids and other collection (fuels) and distribution (CO_2_) infrastructures and businesses may be established. Note that depending on the local supply and demand as well as on the available infrastructure very different solutions may emerge, and further that this would put a large number of people in a position where they actively participate in the new energy economy as so-called “prosumers”. Today, this concept is limited to power and eventually heat, but in the future it may be applicable to fuels as well.

Local generation of chemical energy carriers is especially attractive in regions with underdeveloped infrastructure. Difficult to reach islands and other remote locations could establish an autonomous supply of all end energy carriers needed avoiding transport over large distances, sometimes with enormous efforts regarding energy and cost.

If massively deployed worldwide, eventually more fuels could be produced along this vision of “crowd oil” than needed in the remaining non-electrifiable segments of transport and industry, which could then establish a CO_2_-negative generation of a synthetic oil reserve for future use. Admittedly, this sounds very futuristic given today’s enormous daily consumption of fossil fuels, but it may in the long term provide a valuable alternative to carbonation as a CO_2_ sink.

The intermittent availability of renewable power from wind turbines and photovoltaics brings about technical and economic challenges which have to be dealt with. Energy storage may help to improve the match between power availability and the capacity for processing it into the desired fuel. Moreover, variations of the availability of CO_2_ from the capturing system must also be considered. The intermittent supply reduces the capacity utilization and therefore inevitably increases the cost per litre of produced fuel.

Moreover, space requirements and safety issues have to be considered when it comes to implementation of this vision in large cities. The general idea is that local fuel synthesis will target liquid products with a quality as close as possible to drop in standard, and the products of several fuel synthesis units will be collected and upgraded to finished products in small size plants located in commercial zones.

**Fig. 2:**
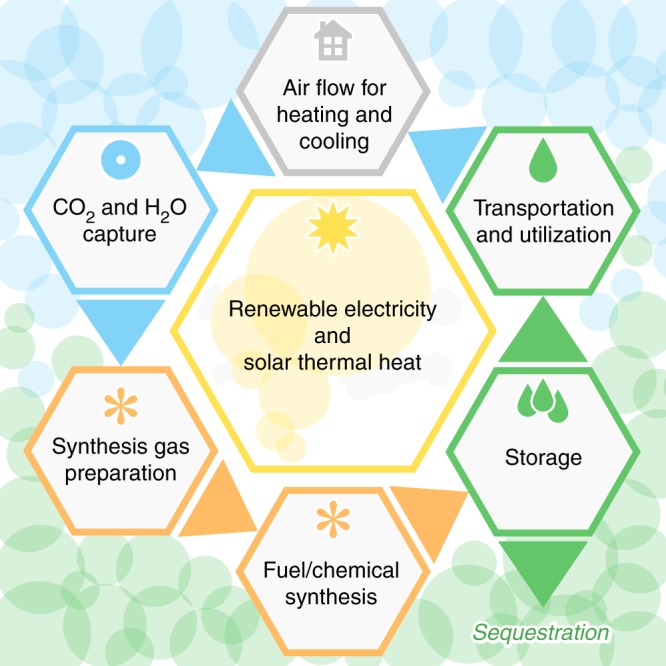
Envisioned modular, on-site renewable hydrocarbon synthesis system based on CO_2_ capture from thin air. Counterclockwise from top: Powered by renewable electricity, using modified A/C and ventilation systems CO_2_ and water can be captured from ambient air. Via electro-, photo- or thermocatalytic processes, synthesis gas is generated which is further converted to hydrocarbon fuels by miniaturized chemical processes such as the Fischer-Tropsch synthesis plus integrated upgrading processes (e.g. hydrocracking) allowing to further increase product yield and quality. The synthesized hydrocarbon fuels can be stored and/or transported for further utilization. In case of combustion for mobility or power generation, in this scenario the CO_2_ emitted is again captured, finally allowing for a closed, net zero carbon cycle, thus, enabling a circular CO_2_ economy based on hundreds, thousands, and ultimately millions of modular plants. When stored without further utilization even negative emissions can be realized

One technical approach to a decentralized synthesis of hydrocarbon fuels based on CO_2_ which appears to be possible already today involves retrofitting air conditioning (A/C) systems in houses, apartment and office buildings with “existing technology” to capture CO_2_ and H_2_O from thin air. Electrolysis of H_2_O can produce H_2_, which combined with captured CO_2_ produces hydrocarbon fuels via Fischer-Tropsch catalysis or related approaches in mass-produced modular conversion systems, illustrated in Fig. 2.

**Fig. 3:**
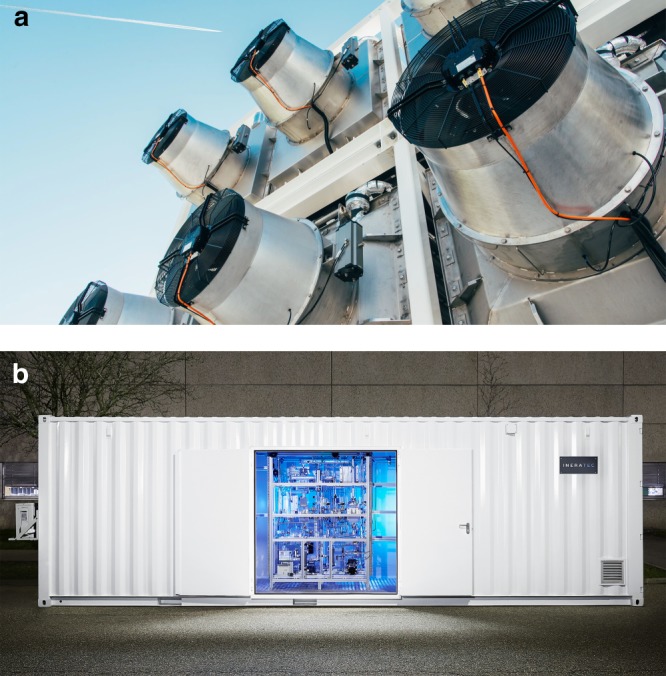
Modular plants for CO_2_ capture and conversion. Commercialized modular systems for CO_2_ capture directly from air (**a**) and for generation of H_2_ by H_2_O electrolysis as well as production of hydrocarbon fuels from CO_2_ and H_2_ (**b**) via compact chemical reactor technology. Copyright permission, Climeworks AG and INERATEC GmbH

It is important to realize that companies have commercialized modular technology (see Fig. 3) for capturing CO_2_ directly from air (DAC) (Climeworks AG, Switzerland; Carbon Engineering, Canada; Skytree, Netherlands), generating H_2_ electrochemically from H_2_O (Siemens AG, Germany; Hydrogenics, Canada; sunfire GmbH, Germany; Proton On Site, USA) or even synthesis gas by further conversion of H_2_ with CO_2_, producing a mixture of H_2_ and CO by co-electrolysis of H_2_O and CO_2_ (sunfire GmbH, Germany), and producing hydrocarbon or oxygenated fuels from CO_2_ and H_2_ or synthesis gas catalytically (INERATEC GmbH, Germany; Gensoric GmbH, Germany; Velocys, USA; Primus Green Energy, USA; Carbon Engineering, Canada; Hydrogenics, Canada; Carbon Recycling International, Iceland).

It would be not that difficult technically to add a CO_2_ capture functionality to an A/C system, and an integrated A/C-DAC unit is expected to show favourable economics. In fact, this has already been patented for lowering the energy requirements for air conditioning in buildings bringing reality to the vision of “crowd oil”^29^.

Anecdotally, A/C is amongst the most useful inventions of the twentieth century. With continued global warming, the prevalence of A/C will likely become more pervasive. Perhaps the use of A/C for making hydrocarbon fuels, if adapted globally as suggested, could be A/C 2.0 of the twenty-first century.

Moreover, in the future direct conversion of solar radiation, CO_2_ and water into hydrocarbon fuels may reach a competitive status compared to the indirect way of first generating power from photovoltaics or concentrated solar energy and then splitting water or CO_2_ by electrolysis. Focused research on photocatalytic solar fuels is underway worldwide, for example at the Joint Center for Artificial Photosynthesis (JCAP) of the California Institute of Technology in Pasadena, CA, USA^11,30,31^, the Helmholtz Center Berlin, Germany, and the University of Toronto, Canada^10,14^, to name only a few of the many institutions active in this field. Technology for direct solar thermal fuels is being developed at the Swiss Federal Institute of Technology (ETH) in Zürich, Switzerland, the German Aerospace Center (DLR) in Cologne, Germany, and the Weizman Institute of Science in Rechovot, Israel (see e.g. special issue on Solar Thermochemistry published in Solar Energy^13^). Again, this list is not complete.

The world’s population currently stands at 7.6 billion people and is growing at the rate of 83 million people per year^32^. Annual estimates of the CO_2_ emission load for all countries in the world place the sum total of anthropogenic CO_2_ emissions in the range of 40 billion metric tons^33^.

Given information on the number of houses, apartment blocks and office buildings in these countries that are, or could be, outfitted with A/C systems, it should be possible to appraise the associated greenhouse gas reduction potential and assess the global capacity to produce, store, distribute, use and recycle renewable hydrocarbon fuels in this way. Moreover, a sustainability assessment based on life cycle analysis and life cycle costing should be capable of providing the necessary insight into the mass, energy and economic flows for such a distributed social technology and sustainability project.

## Renewable synthetic oil well—illustrative examples

In office, commercial and public buildings, a significant volume flow of air is steadily circulated for ventilation and cooling or heating purposes. A typical volumetric exchange rate for office buildings is 5−10 times per hour. Although the concentration of CO_2_ in the air is low, the absolute amount of CO_2_ in contact with the A/C device can be substantial.

**Fig. 4:**
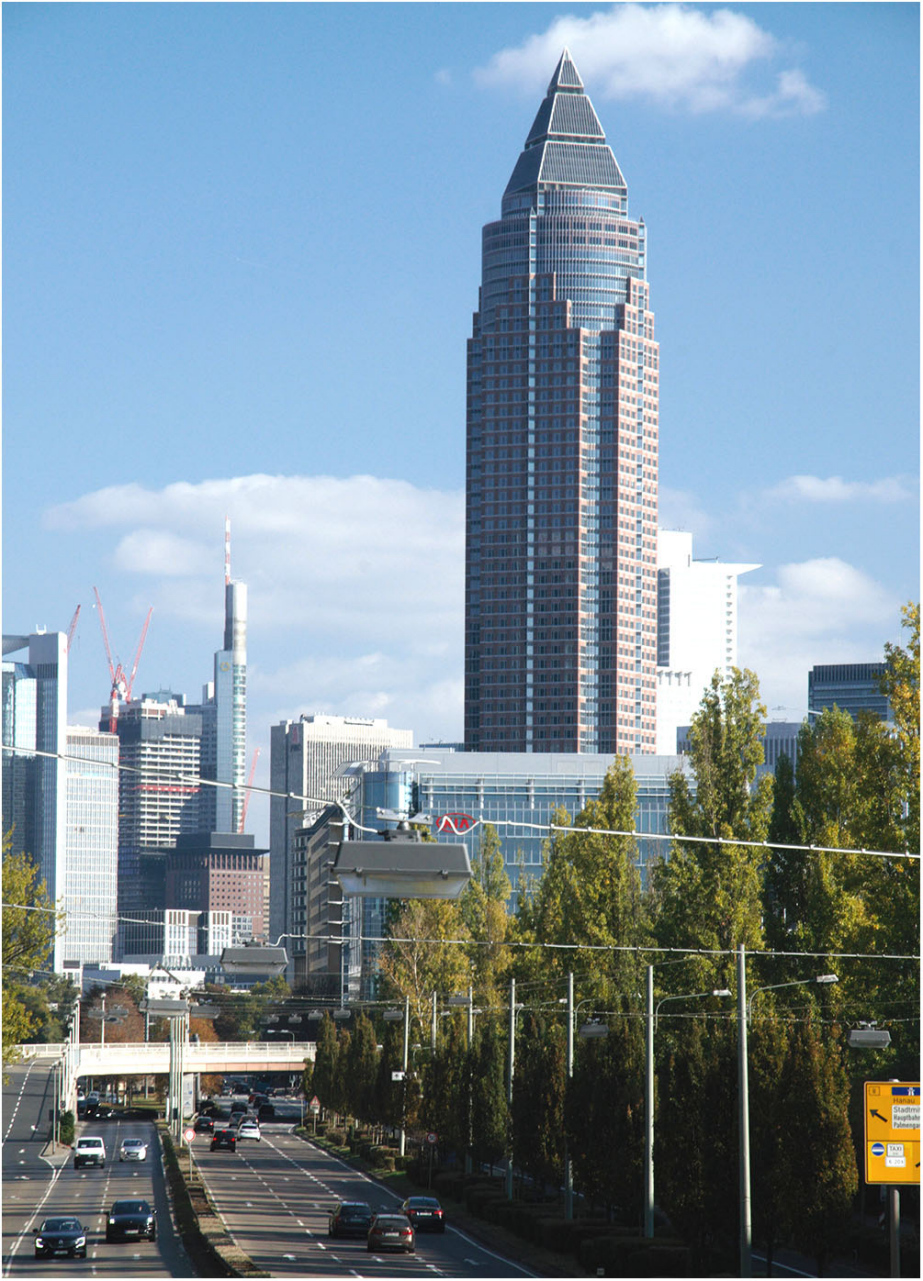
The Frankfurt Fair Tower. Copyright permission by Gerd Bezner (2018)

As an example, one of the landmark buildings in Frankfurt am Main, Germany, its Fair Tower (see Fig. 4), offers 63,000 m^2^ of office space. Assuming 3 m ceiling height yields a total volume of ca. 200,000 m^3^. Based on the above-mentioned recommended ventilation rate for office buildings, this gives an estimated airflow of 1–2 M m^3^ h^−1^. With 400 ppm CO_2_ in air^33^, this translates to 0.75–1.5 t CO_2_ h^−1^ ready for capture. According to recently published data for capturing CO_2_ from air, the energy demand per metric ton of captured CO_2_ is about 1.43 MWh heat and 0.37 MWh electric power^34^. If combined with an air conditioning system, the demand for additional electric power would be substantially lower because only the additional pressure drop of the CO_2_ absorber would have to be covered. Thus, mainly an additional heat input of 1.07–2.15 MW would be required if all the CO_2_ in the contacted air would be recovered. The temperature level needed is 100 °C, which is compatible with solar heat, district heating or reaction heat from the following exothermic fuel synthesis step, e.g., Fischer-Tropsch synthesis.

Pertinent are recent results by the Karlsruhe Institute of Technology (KIT), Climeworks AG, sunfire GmbH, and INERATEC GmbH from the ongoing flagship project “Power-to-X”^35^ which is part of the so-called “Kopernikus-Initiative” of the German Federal Ministry for Education and Research BMBF to support the Energy Transition. They report a containerized plant integrating CO_2_ capture from thin air, high-temperature co-electrolysis of CO_2_ together with steam to produce synthesis gas, and an ultra-compact two-stage fuel synthesis based on the low-temperature Fischer-Tropsch route and hydrocracking, would produce 344 kg of liquid hydrocarbon fuels and 24 kg of wax per metric ton of CO_2_ captured. Carbon efficiency is high due to the recycling of the gaseous product fraction after the fuel synthesis back into the co-electrolysis. The recycle stream mainly contains unreacted CO_2_, CO and H_2_ as well as some CH_4_ and minor amounts of C_2_- to C_4_-hydrocarbons formed in the synthesis as side products. Due to the presence of some inert gas in the feed, a small part of the recycle stream must be discharged. However, even in the worst case studied, per metric ton of CO_2_ captured, at most 80 kg of the recycle stream had to be discharged which corresponded to a loss of carbon of only 10.1%. Hence, the carbon efficiency of the integrated process ranges between 89.9% and close to 100%.

These numbers have been derived from the experimental performance of the individual units together with a process simulation for the integrated process within the detailed engineering of an experimental proof-of-concept plant with a design throughput of 1.25 kg h^−1^ of CO_2_. Moreover, an overall energy efficiency of 50–60% has been determined for the integrated process at a scale between 100 kW and 10 MW for the co-electrolysis unit. Key to the high overall energy efficiency is the utilization of the reaction heat of the Fischer-Tropsch synthesis for providing the steam for the co-electrolysis, which is enabled by the advanced reactor technology used. Based on these data, the amount of CO_2_ potentially captured by the Frankfurt Fair Tower corresponds to a production rate of liquid hydrocarbon fuels of 250–500 kg h^−1^ or 2000–4000 metric tons per year, which needs 40−80 microstructured Fischer-Tropsch synthesis modules or 5−10 containers of INERATEC’s current design as well as the matching co-electrolysis units. Note that the same calculation assuming the whole available office area in Frankfurt am Main, Germany, which is 11.59 million square metres^36^, would be equipped with this technology results in a tentative potential production rate of 370,000–740,000 metric tons per year. Accordingly, for the five cities with the largest office space in Germany together one obtains 2.4–4.8 M metric tons per year.

Moreover, considering the 25,000^36^ grocery stores of just the three biggest players of the German food retailing industry (with an approx. average area of 1200 m^2^ ^36^), by ventilation (heating and air conditioning) approx. 10,000 m^3^ h^−1^ ^37^ and for the rooftop condensers of the cooling system for chillers and refrigerators approx. 40,000 m^3^ h^−1^ ^37,38^, in total approx. 50,000 m^3^ h^−1^ of air circulates within each individual store. This corresponds to a CO_2_ amount of 40 kg h^−1^ to be captured and converted into 14 kg h^−1^ of liquid hydrocarbon fuels, which can be dealt with in a containerized plant. Having a huge scaling effect provided by tens of thousands of stores, each equipped with a number of synthesis units and the matching co-electrolysis units within one or more compact containers, implies that altogether about 1000 metric tons of CO_2_ per hour, corresponding to 350 metric tons CO_2_ per year per store could be captured for processing into hydrocarbon fuels. Impressively, using on-site conversion this would allow provision of 3 M metric tons of hydrocarbon fuels per year, which is about 8% of Germany’s total consumption of diesel of 38.7 M metric tons or 30% of its total consumption of kerosene of 10 M metric tons^39^.

**Fig. 5:**
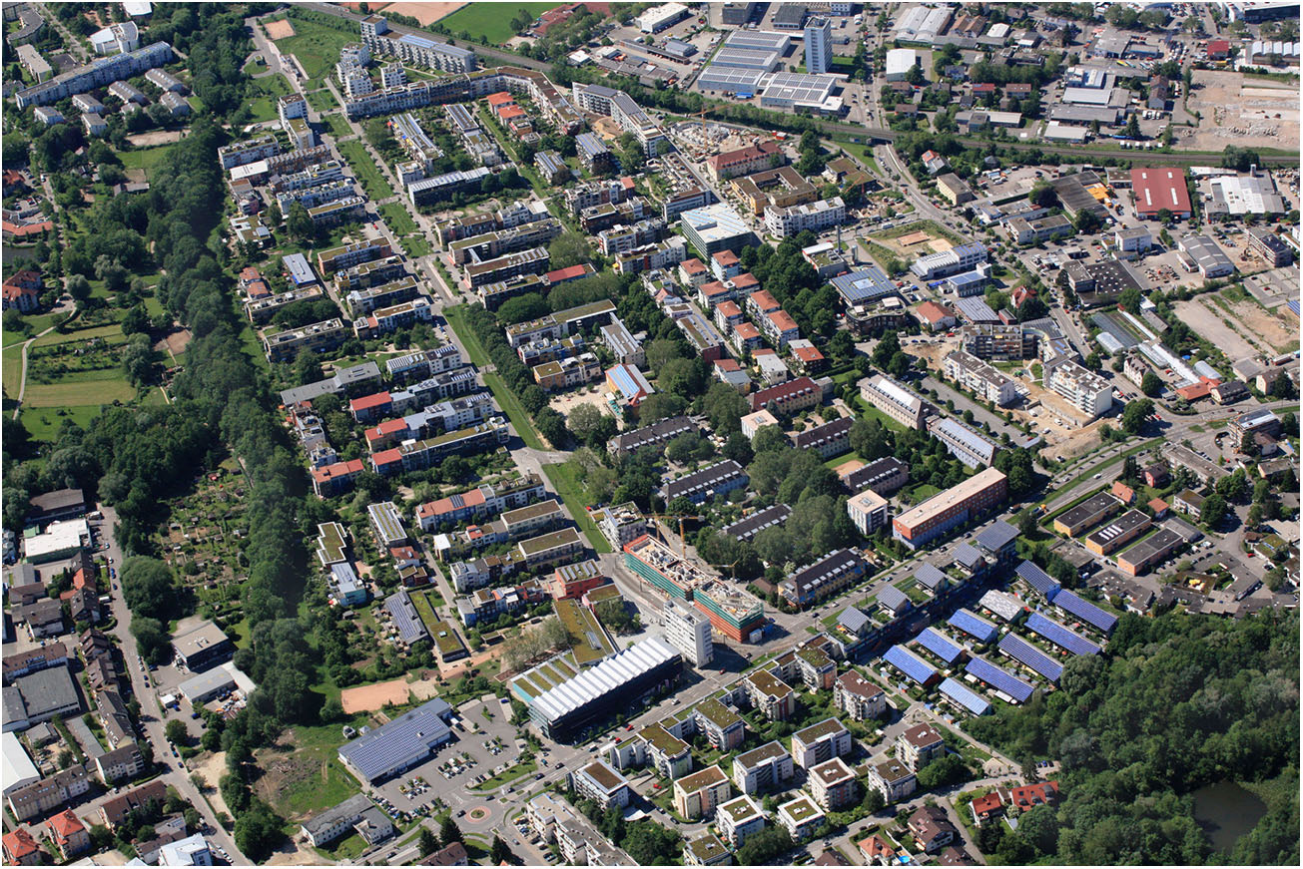
Aerial view of Vauban neighbourhood in Freiburg, Germany. Copyright permission by Erich Meyer, Hasel|Stadt Freiburg (2012)

And what about miniaturized versions? Very small-scale residential CO_2_ capture and conversion units, like for instance developed in the framework of the willpower-energy project funded by the EU Horizon 2020 Programme and guided by Gensoric GmbH^40^, could profitably be implemented in low-energy houses, which technically need ventilation to fulfil hygiene-standards (humidity and odours), because their highly insulated walls and windows don’t allow sufficient air exchange. Especially interesting is the implementation in neighbourhoods with a high share of low-energy houses like for instance the Vauban neighbourhood in Freiburg, Germany (see Fig. 5). For a 70 m^2^ flat (average for one flat in Vauban^41^) with one bathroom, one extra WC, one kitchen and a cellar room a minimum rated airflow of 140 m^3^ h^−1^ can be estimated following the national standard of DIN1946-6^42^. With 5−6 flats per house (average in Vauban^41^) and 400 ppm CO_2_ in ambient air^33^, one building could capture at most 0.5 kg of CO_2_ per hour which, converted to hydrocarbons, would yield 4−5 kg fuel daily. The power required at the electrolysis unit in that scale is in the range of 4–5 kW. System integration, very compact design, low cost and fully automated operation as well as long-term stability and robustness would be imperative. An alternative to individual production units would be a crowd-owned larger production facility in the neighbourhood. All 354 buildings in Vauban^41^ together would for instance capture approx. 200 kg h^−1^ CO_2_, which converted in one to two containers of INERATEC’s current design translates to approx. 620 metric tons of hydrocarbon fuel per year. The 5500 inhabitants in the futuristic and ecological neighbourhood Vauban own 1146 cars^41^. Admittedly this is way less than the average in Germany, where more than 46 million cars^43^ are owned by a bit more than 82 million people^32^. But assuming a consumption of 6 L hydrocarbon fuel per 100 km, each of the cars in Vauban could drive 11,000 km per year with the 620 metric tons of fuel synthesized by the crowd’s own production facilities, which is close to the average mileage of a car in Germany (approx. 14,000 km per year^44^).

These three different examples illustrate the potential of A/C systems equipped with CO_2_ capture to serve as a carbon source for production of a relevant amount of hydrocarbon fuels. At the same time, one can easily calculate that for substitution of the total current refinery output in Germany of 106 M metric tons^39^ based on air-captured CO_2_, about 455 × 10^12^ m^3^ of air would have to be contacted if all the CO_2_ could be removed. Given the total surface area of Germany of ca. 358 × 10^9^ m^2^ the latter translates into a height of 1300 m of air all over Germany that would have to be processed during the course of 1 year. This sounds very large, but per day it is approximately 3.5 m “only” which may appear less threatening. Yet as 100% removal is certainly unrealistic the actual values would be larger.

One may argue that biomass is a more appropriate source of carbon than thin air given the high dilution of the CO_2_ in air. However, if the amount of carbon needed for 106 M metric tons of oil products had to be supplied by biomass grown within the German borders, the required volume of air per land area would not be lower. In that case, the growing plants would have to extract the CO_2_ from the air and only a fraction of that CO_2_ would be found in the final products.

On a side note, around 302 × 10^9^ m^3^ of air per year are passed through the lungs of the whole German population for providing the oxygen to drive their metabolism (derived from 10 m^3^ per day and person). This is about three orders of magnitude less compared to the volume of air to be processed in order to provide the carbon to fuel their vehicles and produce the chemicals they use.

The renewable electrical energy needed to synthesize 106 M metric tons of oil products from CO_2_ is huge. Assuming a conversion efficiency from electrical energy to fuel of 50%, it would be in the range of 2.800 TWh. Today, about 146 TWh of renewable energy^45^ is harvested per year from all the wind turbines and solar panels installed in Germany. There is certainly potential for increasing this value, but the amount of renewable electrical energy required is an enormous challenge. According to statistical data, 9.1% of all land area in Germany is covered by buildings^46^ and an annual average solar radiation of 1050 kWh a^−1^ m^−2^ is measured^47^. With a panel efficiency of 15% roughly half of the whole rooftop area would have to be covered by solar panels to generate 2800 TWh per year.

In summary, these numbers tell us that the overall consumption of oil products must be reduced to the minimum needed. Furthermore, air-captured CO_2_ is a relevant carbon source, which should be considered. And finally, viable mechanisms have to be identified and implemented for quickly building up new infrastructure for decentralized conversion of renewable electrical energy into hydrocarbon fuels.

While the numbers here are for one highly industrialized country mainly for illustration, it is not proposed to strive for energy autonomy on national level both for technical, economic and political reasons. For the case of Germany, there are other countries around in Europe where the potential for wind and solar energy is higher or A/C systems are used more widely, and this equally holds for other continents. Moreover, fair trade of renewable energy and CO_2_ provides opportunities for economic development and is therefore important for the global economy.

## Towards a multifaceted new energy economy

Depending on the technology, the hydrocarbon fuels produced in small, decentralized plants may need a final workup to match the technical specifications for use. This could be done locally in new, dedicated “mini-refineries” or centrally in some of the existing refineries. Given the high-energy density, liquid hydrocarbons can easily be transported by railways, trucks, and ships. When the capacity of individual wells reaches a threshold, they could also be connected with pipelines. When there is not enough power from renewables over longer periods of time, and electrochemical storage is not sufficient to bridge that time the fuels could be back-converted into electricity in fuel cells or other decentralized power-generation units integrated into an intelligent power grid. Note that this guarantees security of supply but comes at the price of a much lower round-trip efficiency compared to storage in batteries.

Depending on the participation schemes and business models adopted, the fuels could help to satisfy the energy requirements, such as transportation, of the inhabitants of homes, apartments, offices, commercial and public buildings, with any excess fed into storage and distribution systems, in exchange for payments to the property owner, analogous to the feed-in-tariff programmes that kick-started the photovoltaic industry globally.

Once this approach is broadly implemented and when the production of renewable hydrocarbon fuels for transport and chemical industry exceeds its consumption, they can be stored on a large scale, for example in the volumes of depleted oil wells, coalmines and salt caverns. This paradigm envisions an intriguing method of banking synthetic fuels for future energy needs.

On a cautionary note, just as there is not much of a public appetite for enabling massive underground CO_2_ capture and storage, the same attitude may apply to storing renewable hydrocarbon fuels in empty spaces, such as depleted oil wells. Safety and environmental issues such as possible contamination of water supplies would certainly have to be investigated.

Since the industrial revolution, energy companies have provided gas, oil, coal and electricity to a passive population and their healthy profits have attracted large investors in the insurance and pension industries. These profits are now under threat by price caps and carbon taxes imposed by governments, and by the continued decrease in the cost of renewable power, exacerbated by the changing behaviour of a more active population towards sustainability.

The generation of power, once the reserve of the fossil-based energy companies, is beginning to shift to people, through “crowd electricity”. This is a global project involving the increasing adoption of solar panels and wind turbines, on the roofs of houses and in open fields, with excess electricity either being stored in lithium ion batteries or fed into the electricity grid.

“Crowd oil” could also serve as a global project motivated by the health and well-being of a growing population of global shareholders with a common bond and mutual interest in a sustainable future. This presents an alternative to relying upon government and industrial incentives, often skewed towards delivering financial returns to their corporate executives, directors and shareholders rather than promoting public and environmental health.

Implementation of a grid of renewable synthetic oil wells with private production offers an opportunity to transform an asymmetric global fossil energy resource of “have and have not” countries, with its associated geo-political, geo-social and geo-economic problems, into a more symmetrical one, wherein all countries can become energy independent in a “people friendly”, less complicated circular and sustainable economy.

It is an important part of this vision that the production of renewable fuels from carbon dioxide will be integrated as far as possible into the existing built environment, i.e. limiting the additional land use. This means that buildings populated by humans will act like inhabited technical photosynthesis systems, which is a very intriguing idea given the ever-growing number of human beings on this planet.

## Outlook

The preliminary analysis presented above demonstrates the potential of capturing CO_2_ from air conditioning systems in buildings, for making a substantial amount of liquid hydrocarbon fuels. While the analysis considers the CO_2_ reduction potential, carbon efficiency and overall energy efficiency, it does not touch on spatial, or economic metrics for the requisite systems. These have to be obtained from a full techno-economic and life cycle analysis of the entire system. However, with the perspective of mass fabrication of modular intensified units empowered by digitalization and additive manufacturing, the economics as well as the space requirements could well become feasible. Moreover, the long-term reliability and stability of all components of the proposed fully integrated systems have to be addressed in depth by future engineering research in order to prove the feasibility of the concept.

The vision of empowering people with a technology to become energy independent while contributing to a circular economy, a “social energy autarky”, gives control to the people to help solve the greenhouse gas, global warming and climate change problems themselves rather than having to rely on the power-generating industries to rise to the challenge. In order to make this happen, social sciences are needed to investigate how individuals and organizations forming the rather diverse societies around the globe could be motivated to take collective action against global warming and support the implementation of the vision of “crowd oil”. As a side note, while less radical, the World Energy Council recently suggested with its report on a power-to-X roadmap the establishment of an economic and political framework for the fast worldwide establishment of power-to-X technologies^48^.

## Data Availability

All data generated or analysed during this study are included in this published article.
